# Chronic Pain and Pain Tolerance in Psoriasis: A Cross-sectional Study of Three Population-based Cohorts in the Netherlands and Norway

**DOI:** 10.2340/actadv.v106.adv-2026-0510

**Published:** 2026-06-01

**Authors:** Juliette Bollemeijer, Christian Page, Isabel Klein, Kjersti Danielsen, Anne-Sofie Furberg, Marita Jenssen, Ólöf Steingrimsdottir, Audun Stubhaug, Lieke Kuiper, Joyce van Meurs, Tamar Nijsten, Christopher Nielsen, Luba Pardo

**Affiliations:** 1 Department of Dermatology, Erasmus MC University Medical Center, Rotterdam, The Netherlands; 2 Department of Physical Health and Ageing, Norwegian Institute of Public Health, Oslo, Norway; 3 Department of Clinical Medicine, UiT The Arctic University of Norway, Tromsø, Norway; 4 Department of Microbiology and Infection Control, University Hospital of North Norway, Tromsø, Norway; 5 Department of Health and Social Sciences, Molde University College, Molde, Norway; 6 Department of Dermatology, University Hospital of North Norway, Tromsø, Norway; 7 Department of Research, Oral Health Centre of Expertise in Eastern Norway, Oslo, Norway; 8 Institute of Clinical Medicine, University of Oslo, Oslo, Norway; 9 Department of Pain Management and Research, Oslo University Hospital, Oslo, Norway; 10 Department of Internal Medicine, Erasmus MC University Medical Center, Rotterdam, The Netherlands; 11 Department of Chronic Diseases, Norwegian Institute of Public Health, Oslo, Norway

Pain is increasingly recognized as an important but under-addressed symptom in psoriasis. Although often attributed to psoriatic arthritis (PsA) or skin-related pain, individuals with psoriasis or PsA also report pain in other musculoskeletal structures, including axial and buttock pain (1, 2). Quantitative sensory testing (QST) uses standardized experimental stimuli to assess pain thresholds and tolerance, and small studies in psoriasis or PsA have reported reduced pressure pain thresholds (3, 4). However, large population-based studies assessing chronic pain and QST measures in psoriasis, both with and without PsA, are lacking, despite the substantial impact of pain in psoriasis on health-related quality of life (5). Using data from three large population-based cohorts in the Netherlands and Norway, we therefore examined whether individuals with psoriasis differ from controls in chronic pain, number of pain sites, pain intensity and pain tolerance assessed by 2 QST measures: the cold pressor test (CPT) and cuff pressure algometry (PAlg). We also evaluated the influence of PsA, psoriasis activity and severity on these associations.

## METHODS

### Study population

**Fig. 1.**
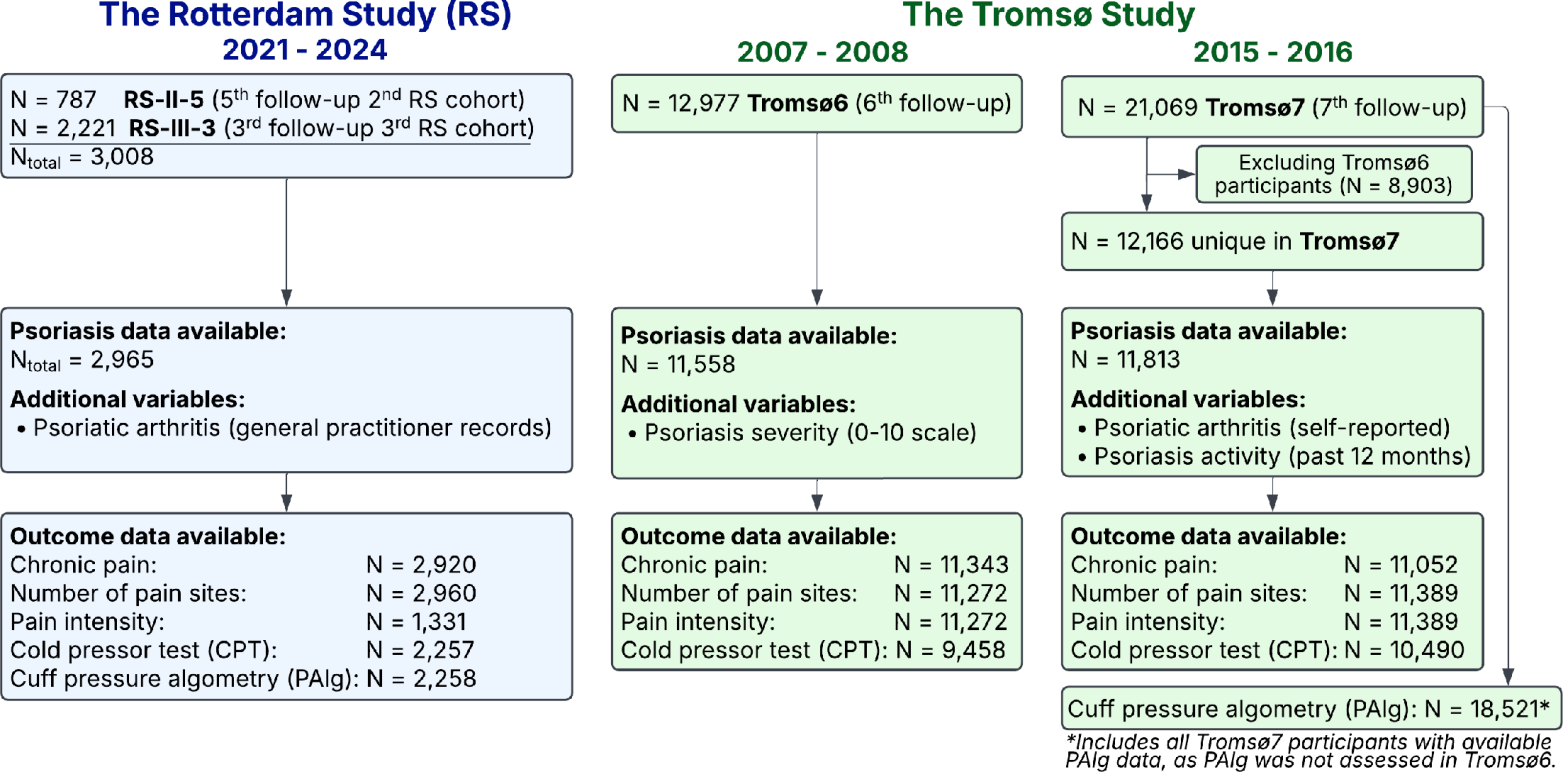
Flow diagram of participant inclusion and availability of psoriasis and pain-related data in the Rotterdam Study and the Tromsø Study cohorts.

This cross-sectional study included participants from the population-based Rotterdam Study (RS; the Netherlands) and Tromsø Study (Tromsø6 and Tromsø7; Norway) (6–8). Participants with psoriasis data were included: RS *n*=2,965 (2021–2024), Tromsø6 *n*=11,558 (2007–2008) and Tromsø7 *n*=11,813 (2015–2016; Fig. 1). For outcomes absent in Tromsø6, the full Tromsø7 cohort was used (*n*=21,069).

### Psoriasis and psoriatic arthritis

In the RS, physician-diagnosed psoriasis was self-reported and validated using general practitioner (GP) records, which were also screened for PsA. In Tromsø, psoriasis was defined as self-reported ever-psoriasis or physician-diagnosed psoriasis; PsA was self-reported in Tromsø7 only. Psoriasis severity in Tromsø6 ranged from 0 (no symptoms) to 10 (most severe symptoms). In Tromsø7, psoriasis activity was self-reported as psoriasis rash during the past 12 months (exact definitions in Table SI).

### Pain outcomes and quantitative sensory testing

Chronic pain was defined as pain lasting ≥3 months with ≥1 pain site and pain intensity ≥2 on a numeric rating scale (NRS). Pain intensity was assessed separately using the same NRS (0–10). Pain-site counts came from cohort-specific body maps or region lists; as “skin” and “other” sites were recorded only in Tromsø6, they were excluded from the main analyses.

CPT was performed in all cohorts using hand immersion in 3 °C water; maximum duration was 121 s in RS, 106 in Tromsø6 and 120 in Tromsø7. PAlg was conducted in RS and Tromsø7, with calf cuff inflation at 1  kPa/second to a maximum of 100  kPa or until intolerable pain; termination pressure was recorded as pain tolerance (Table SI).

### Statistical analyses

Categorical variables are presented as frequencies with percentages, and continuous variables as medians with interquartile ranges (IQR) after Shapiro–Wilk testing.

#### Main analyses

Associations between psoriasis and pain outcomes (chronic pain, number of pain sites, pain intensity) and QST outcomes (CPT, PAlg) were assessed with multivariable regression adjusted for age, sex, body mass index, education and smoking. Logistic regression was used for chronic pain, ordinal logistic regression for pain intensity, zero-inflated Poisson regression for number of pain sites and Cox proportional hazards models for QST outcomes (time-to-termination), with test termination as event and right-censoring at maximum limits.

Missing data were handled by listwise exclusion. RS analyses were performed using IBM SPSS Statistics 28.0.1.0, and Tromsø analyses in R 4.2. Pooled estimates were obtained using random-effects meta-analysis with the metafor package in R, weighted by cohort sample size (9).

#### Sensitivity analyses

Sensitivity analyses excluded participants with PsA in the RS and Tromsø7 (PsA was not assessed in Tromsø6; full Tromsø7 used). Additional analyses examined psoriasis activity in Tromsø7 with and without PsA, psoriasis severity in Tromsø6, GP-validated psoriasis cases versus controls in the RS, inclusion of skin pain as a pain site in Tromsø6, and additional adjustment for analgesic use before QST.

## RESULTS

Population characteristics are described in Table SII. In the main pooled meta-analyses, psoriasis was associated with chronic pain (odds ratio (OR): 1.28, 95 % confidence interval (CI): 1.17–1.40), more pain sites (incidence rate ratio (IRR): 1.28, 95% CI: 1.13–1.46), and higher pain intensity (OR: 1.24, 95% CI: 1.12–1.38; Table SIII). No significant associations were found with QST; PAlg showed a hazard ratio (HR) of 1.00 (95% CI: 0.95–1.05), while CPT showed a trend (HR: 1.12, 95% CI: 0.96–1.30).

After excluding participants with PsA, most associations attenuated, but psoriasis remained associated with more pain sites (IRR 1.08, 95% CI 1.02–1.15). In Tromsø7, psoriasis activity showed stronger associations than in the main analyses with chronic pain (OR: 1.46, 95% CI: 1.23–1.72), pain sites (IRR: 1.41, 95% CI: 1.30–1.52), pain intensity (OR: 1.29, 95% CI: 1.12–1.48), and reduced pain tolerance measured by CPT (HR: 1.11, 95% CI: 1.00–1.23); PAlg remained non-significant (HR: 0.99, 95% CI: 0.93–1.04). In Tromsø6, higher psoriasis severity showed a similar pattern (not shown). After excluding PsA from the Tromsø7 psoriasis activity analyses, the association with pain sites persisted (IRR: 1.17, 95% CI: 1.05–1.29). Sensitivity analyses restricted to GP-validated psoriasis cases strengthened associations in the RS, whereas including skin pain as a site in Tromsø6 and additionally adjusting for recent analgesic use prior to QST did not substantially change the estimates (not shown).

## DISCUSSION

In this study of 26,336 adults, psoriasis was associated with chronic pain, more pain sites, and higher pain intensity, particularly in active or more severe disease. Most associations attenuated after excluding PsA, supporting PsA as a major driver of pain in psoriatic disease. However, an association with the number of pain sites persisted. This may reflect undiagnosed or early PsA, a common clinical challenge (10, 11). Neuroimmune mechanisms may also contribute, as psoriasis-related cytokines can influence nociceptive processing and pain sensitization, overlapping with mechanisms involved in widespread pain conditions such as fibromyalgia (12). While fibromyalgia is well described in PsA, its relationship with psoriasis without PsA remains insufficiently understood (13).

Because pain was assessed independently of psoriasis, reported pain likely reflects general pain perception. Including “skin pain” as a pain site in Tromsø6 did not materially change estimates, possibly because psoriasis patients often describe skin-related sensations such as burning or aching without labelling them as “pain” unless prompted (14).

Reduced cold pain tolerance observed in individuals with active psoriasis was modest and diminished after PsA exclusion, indicating that PsA-related pain mechanisms likely influenced QST findings. This aligns with reduced cold pain tolerance reported in other inflammatory arthritides, such as rheumatoid arthritis (15).

Several limitations should be considered. Psoriasis was primarily self-reported, cross-cohort assessments differed, and data on PsA, psoriasis activity, and severity were not available in all cohorts. Participants were predominantly middle-aged or older, which may limit generalizability to younger populations despite adjustment for age. The cross-sectional design precludes causal inference. Strengths include the large population-based sample from two countries including the full spectrum of psoriasis severity and the combined assessment of pain and QST outcomes. Overall, psoriasis was associated with adverse pain outcomes, largely explained by concomitant PsA, whereas the association with number of pain sites persisted after exclusion of known PsA. Longitudinal studies are needed to clarify temporality and whether the number of pain sites predicts incident PsA.

## Data Availability

Due to restrictions based on privacy regulations and informed consent of the participants, data cannot be made freely available in a public repository. However, data may be obtained upon request from the Rotterdam Study and the Tromsø Study. Requests for data from the Rotterdam Study should be directed to the management team of the Rotterdam Study (datamanagement.ergo@erasmusmc.nl), which has a protocol for approving data requests. Data from the Tromsø Study may be requested via application to the Tromsø Study Data and Publication Committee. Contact information: The Tromsø Study, Department of Community Medicine, Faculty of Health Sciences, UiT The Arctic University of Norway; e-mail: tromsous@uit.no.
