# Supplementary material for: Chronic Pain and Pain Tolerance in Psoriasis: A Cross-sectional Study of Three Population-based Cohorts in the Netherlands and Norway
Source: Acta Derm Venereol. 2026 Jun 1;106:0510. doi: 10.2340/actadv.v106.adv-2026-0510 (PMC13224780; doi:10.2340/actadv.v106.adv-2026-0510)
Supplement: Supplementary file 1. [file ActaDv-106-0510-s0001.pdf]

Table SI. Cohort-specific definitions and measurements of psoriasis, pain, and quantitative sensory testing variables.

Definition	Rotterdam Study	Tromsø6	Tromsø7
**Psoriasis**			
**Self-reported psoriasis**	-	"Do you have, or have you ever had psoriasis?"	"Do you have, or have you ever had psoriasis?"
**Self-reported physician-diagnosed psoriasis**	"Have you ever been diagnosed with psoriasis by a physician?"	"Have you ever been diagnosed with psoriasis by a physician?"	"Have you ever been diagnosed with psoriasis by a physician?"
**Validation in GP records**	GP records reviewed for self-reported psoriasis cases. Definite case: diagnosis by dermatologist or rheumatologist, or ≥2 GP entries documenting psoriasis. Possible case: 1 GP entry or diagnosis by non-specialist. Control: self-reported "no" psoriasis. Missing: no consent to GP record access.	-	-
**Psoriatic arthritis (PsA)**	GP records also reviewed for PsA: diagnosis by any specialist or ≥1 GP entry documenting PsA	-	"Do you have, or have you ever had psoriatic arthritis?"
**Psoriasis severity**	-	"On a scale from 0 (no disease symptoms) to 10 (most severe disease symptoms), how severe is your psoriasis today?"	-
**Psoriasis activity in past 12 months**	-	-	"Have you had a psoriasis rash within the last 12 months?"
**Pain**			
**Chronic pain**	Pain ≥3 months, ≥1 pain site and NRS ≥2	Pain ≥3 months, ≥1 pain site and NRS ≥2	Pain ≥3 months, ≥1 pain site and NRS ≥2
**Number of pain sites**	Body map; 68 regions (34 front, 34 back)	Checklist of 15 body regions	Graphical Index of Pain; 10 body regions
**Pain intensity**	Average pain intensity, NRS 0-10	Average pain intensity, NRS 0-10	Average pain intensity, NRS 0-10
**Quantitative sensory testing**			
**Cold pressor test (CPT)**	Hand immersion (including wrist) in 3°C water; maximum 121 seconds; right-censored at 119 seconds to standardize technician variation	Hand immersion (including wrist) in 3°C water; maximum 106 seconds	Hand immersion (including wrist) in 3°C water; maximum 120 seconds
**Cuff pressure algometry (PAlg)**	Calf cuff inflated at 1 kPa per second using a computerized cuff algometer to a maximum of 100 kPa (100 seconds of inflation) or until intolerable pain. Pressure at test termination was recorded as pain tolerance. Second-leg tolerance used for analyses.	-	Calf cuff inflated at 1 kPa per second using a computerized cuff algometer to a maximum of 100 kPa (100 seconds of inflation) or until intolerable pain. Pressure at test termination was recorded as pain tolerance. Second-leg tolerance used for analyses.

Abbreviations: GP, general practitioner; NRS, numeric rating scale; PAlg, cuff pressure algometry; PsA, psoriatic arthritis.

Table SII. Population characteristics of participants of the Rotterdam Study and the Tromsø Study (Tromsø6 and Tromsø7).

Variable	Subcategory Rotterdam Study	Rotterdam Study (RS-II & RS-III) (n = 2,965)	Subcategory Tromsø Study (if different)	Tromsø6 (n = 11,558)	Tromsø7 (n = 11,813)
**Age in years†, *median (IQR)***		73 (68 – 78)		59 (46 – 66)	52 (45 – 60)
**Sex, *n (%)***	**Male**	1258 (42.4%)		5467 (47.3%)	5703 (48.3%)
	**Female**	1707 (57.6%)		6091 (52.7%)	6110 (51.7%)
**BMI in kg/m^2††, *median (IQR)***		27 (25 – 30)		26 (24 – 29)	27 (24 – 30)
**Education level†††, *n (%)***	**Low education**	260 (8.8%)	**Primary**	3098 (26.8%)	2032 (17.2%)
	**Medium education**	1691 (57.0%)	**Technical**	2962 (25.6%)	NA
	**High education**	986 (33.3%)	**High school**	854 (7.4%)	3203 (27.1%)
			**College or university (< 4 years)**	2105 (18.2%)	2385 (20.2%)
			**College or university (≥ 4 years)**	2403 (20.8%)	4079 (34.5%)
	Missing	*28 (0.9%)*	***Missing***	*136 (1.2%)*	*114 (1.0%)*
**Smoking status‡, *n (%)***	**Never smoker**	1021 (34.4%)	**Never smoker**	4150 (35.9%)	4553 (38.5%)
	**Former smoker**	1718 (57.9%)	**Former smoker**	4416 (38.2%)	5741 (48.6%)
	**Current smoker**	225 (7.6%)	**Occasional smoker**	1687 (14.6%)	462 (3.9%)
			**Daily smoker**	1154 (10.0%)	1053 (8.9%)
	Missing	*1 (0%)*	***Missing***	*151 (1.3%)*	*4 (0%)*
**Self-reported psoriasis‡‡, *n (%)***			**General self-reported psoriasis**	1267 (11.0%)	1227 (10.4%)
	**Self-reported physician-diagnosed psoriasis**	178 (6.0%)	**Self-reported physician-diagnosed psoriasis**	1086 (9.4%)	943 (8.0%)
			**Self-reported psoriasis. not physician-diagnosed**	181 (1.6%)	284 (2.4%)
	**No self-reported psoriasis**	2787 (94.0%)	**No self-reported psoriasis**	10291 (89.0%)	10586 (89.6%)
	Missing	*0 (0%)*	***Missing***	*0 (0%)*	*0 (0%)*
**Psoriasis severity‡‡‡, *median (IQR)***		NA		3 (1 – 5)	NA
	Missing		***Missing***	*363 (28.7%)*	
**Psoriasis rash in last 12 months§, *n (%)***	**Yes**	NA		NA	693 (5.9%)
	**No psoriasis rash in last 12 months**				534 (4.5%)
	**No self-reported psoriasis**				10586 (89.6%)
**Validated psoriasis in GP record§§, *n (%)***	**Yes, definite psoriasis**	80 (2.7%)		NA	NA
	**Possible psoriasis**	27 (0.9%)			
	**Self-reported psoriasis not validated**	34 (1.1%)			
	**No self-reported psoriasis**	2787 (94.0%)			

	No access to GP record	*37 (1.2%)*			
**Psoriatic arthritis[§§§], *n (%)***	**Mentioned in GP record**	17 (0.6%)	**Self-reported psoriatic arthritis**	NA	319 (2.7%)
	**No or unknown**	124 (4.2%)	***Missing***		*93 (0.8%)*
	No access to GP record	*37 (1.2%)*			
	Not assessed (no self-reported psoriasis)	*2787 (94.0%)*			
**Analgesic use[¶], *n (%)***	**Yes**	416 (14.0%)		778 (6.7%)	1148 (9.7%)
	**No**	1923 (64.9%)		9900 (85.7%)	10608 (89.8%)
	Missing	*626 (21.1%)*	***Missing***	*880 (7.6%)*	*57 (0.5%)*
**Pain**					
**Chronic pain[¶¶], *n (%)***	**Yes**	1311 (44.2%)		3336 (28.9%)	5663 (47.9%)
	**No**	1609 (54.3%)		8008 (69.3%)	5389 (45.6%)
	Missing	*45 (1.5%)*		*214 (1.9%)*	*761 (6.4%)*
**Number of pain sites[¶¶¶], *median (IQR)***		4 (2 – 8)		3 (2 – 5)	2 (1 – 3)
	Missing, n (%)	*0 (0%)*	***Missing, n (%)***	*0 (0%)*	*0 (0%)*
**Pain intensity (NRS)[◊], *median (IQR)***		6 (4 – 7)		5 (4 – 6)	5 (4 – 7)
	Missing, n (%)	*0 (0%)*	***Missing, n (%)***	*0 (0%)*	*0 (0%)*
**Quantitative Sensory Testing**					
**Cold pressor test[◊◊], *n (%)***	**Reached maximum time**	591 (19.9%)		2893 (25.0%)	4063 (34.4%)
	**Stopped before maximum time**	1666 (56.2%)		6565 (56.8%)	6426 (54.4%)
	Missing, n (%)	*708 (23.9%)*	***Missing, n (%)***	*2100 (18.2%)*	*1324 (11.2%)*
**Cuff pressure algometry[◊◊◊], *n (%)***	**Reached maximum pressure**	88 (3.0%)		NA	1138 (5.6%)
	**Stopped before maximum pressure**	2170 (73.2%)			17383 (85.1%)
	Missing, n (%)	*707 (23.8%)*	***Missing, n (%)***		*1907 (9.3%)*

Abbreviations: BMI, Body Mass Index; GP, general practitioner; IQR, Interquartile Range; n, number of participants; NA, not applicable; NRS, Numeric Rating Scale; RS, Rotterdam Study.

†Age at time of pain assessments.

††BMI calculated as weight (kg)/height squared (m^2).

††† Education categories differ between cohorts. The Rotterdam Study categories include: Low = primary education; medium = lower vocational education, lower secondary education, or intermediate vocational education; high = general secondary education, higher vocational education, or university. Tromsø Study categories are listed in the table.

‡Smoking status refers to self-reported smoking of cigarettes. Tromsø definition: “occasional smoker” = smokes sometimes but not daily; “daily smoker” = smokes daily. If both items were marked “yes”, participants were classified as daily smokers.

‡‡ Self-reported psoriasis differed between studies. In the Rotterdam Study, self-reported physician-diagnosed psoriasis was captured through: “Have you ever been diagnosed with psoriasis by a physician?” In the Tromsø Study, general self-reported psoriasis was captured through: “Do you have, or have you ever had psoriasis?”, and self-reported physician-diagnosed psoriasis with: “Have you ever been diagnosed with psoriasis by a physician?”. In the Tromsø analyses, any “yes” (to either item) counted as self-reported psoriasis.

‡‡‡ Tromsø6 participants rated their current psoriasis severity using the question, "On a scale from 0 (no disease symptoms) to 10 (most severe disease symptoms), how severe is your psoriasis today?" The scale ranges from 0 (no symptoms) to 10 (most severe symptoms). The median and interquartile range (IQR) are shown only for participants who self-reported having psoriasis (n = 1267).

§ Tromsø7 participants were asked, "If you have, or have had psoriasis – have you had a psoriasis rash within the last 12 months?"

§§ In the Rotterdam Study, GP records were screened to validate self-reported psoriasis. In Tromsø, no medical-record validation was performed.

§§§ Psoriatic arthritis was GP-recorded in the Rotterdam Study (only among participants with self-reported psoriasis). Psoriatic arthritis was self-reported in Tromsø7.

¶ Analgesic use was defined as the intake of any analgesic within the 24 hours preceding quantitative sensory testing.
¶¶ Chronic pain defined as pain lasting ≥3 months, with ≥1 site marked on the pain drawing and NRS ≥2.
¶¶¶ Number of marked pain sites on the pain drawing of those with pain lasting ≥3 months. In the Rotterdam Study, sites were marked on a standardized body map with 68 regions (34 front and 34 back) based on regular pain during the prior six weeks. In Tromsø6, sites were checked from a 15-location list, including “skin” as a separate category. In Tromsø7, sites were recorded with the Graphical Index of Pain (first tier: 10 regions). The number is presented only for participants with chronic pain (≥3 months), leading to 0% missing data.
◊ Pain intensity measured using an NRS from 0 (no pain) to 10 (worst imaginable pain). Pain intensity is presented only for participants with chronic pain (≥3 months), leading to 0% missing data.
◊◊ Cold pressor test: hand immersion in 3°C water, participants immersed a hand in 3°C water for up to 121 seconds (Rotterdam Study), 106 seconds (Tromsø6), or 120 seconds (Tromsø7). In the Rotterdam Study, to standardize technician-related variation, a uniform right-censoring limit of 119 seconds was applied: withdrawals <119 seconds were coded as events; times ≥119 seconds were censored.
◊◊◊ Cuff pressure algometry: inflatable cuffs applied to the calf; pressure increased at 1 kPa/second to a maximum of 100 kPa (corresponding to 100 seconds). Both legs were tested sequentially, and the second-leg tolerance was used for analysis. Not performed in Tromsø6. In Tromsø7, the full cohort with available psoriasis data (n = 20,428) was used, as there was no overlap with Tromsø6 participants for this test; denominators therefore differ from other Tromsø7 rows.

Table SIII. Associations of psoriasis with pain and quantitative sensory testing outcomes in the main meta-analysis and sensitivity analyses.

			**Main meta-analysis**			**Sensitivity analysis: Excluding PsA**			**Sensitivity analysis: Psoriasis activity**			**Sensitivity analysis: Psoriasis activity excluding PsA**		
			RS, Tromsø6, and Tromsø7			***RS, Tromsø7***[†††]			***Tromsø7***			***Tromsø7***		
**Outcome**	**Analysis method**	**Measure**	**N**[††]	**Effect (95% CI)**	**P**	**N**[††]	**Effect (95% CI)**	**P**	**N**[††]	**Effect (95% CI)**	**P**	**N**[††]	**Effect (95% CI)**	**P**
**Pain**														
Chronic pain	Logistic regression	OR	24269	**1.28 (1.17 – 1.40)**	**<.001**	20441	1.07 (0.91 – 1.26)	0.43	10430	**1.46 (1.23 – 1.72)**	**<.001**	10150	1.09 (0.90 – 1.31)	0.40
Number of pain sites	Poisson regression	IRR	24973	**1.28 (1.13 – 1.46)**	**<.001**	21242	**1.08 (1.02 – 1.15)**	**0.005**	10874	**1.41 (1.30 – 1.52)**	**<.001**	10580	**1.17 (1.05 – 1.29)**	**0.003**
Pain intensity (NRS)	Ordinal logistic regression	OR	23691	**1.24 (1.12 – 1.38)**	**<.001**	19976	1.04 (0.95 – 1.14)	0.43	10874	**1.29 (1.12 – 1.48)**	**<.001**	10580	0.99 (0.85 – 1.16)	0.91
**Quantitative sensory testing**[†]														
Cold pressor test	Cox regression	HR	21853	1.12 (0.96 – 1.30)	0.14	19282	1.03 (0.96 – 1.10)	0.43	9910	**1.11 (1.00 – 1.23)**	**0.04**	9653	1.02 (0.90 – 1.15)	0.75

Abbreviations: CI; confidence interval, HR; hazard ratio, IRR; incidence rate ratio, N; number, NRS; numeric rating scale, OR; odds ratio, P; P-value; PsA; psoriatic arthritis.

Bold indicates statistical significance (P < 0.05). [†]Results for cuff pressure algometry (PAlg) are not presented separately because hazard ratios were consistently close to 1.0 and non-significant in both the main and sensitivity analyses. [††]N denotes the total number of participants included in each model (not limited to psoriasis cases). [†††]In these sensitivity analyses, the full Tromsø7 cohort was used because PsA was not assessed in Tromsø6.
